# Prediction model for pancreatic cancer risk in the general Japanese population

**DOI:** 10.1371/journal.pone.0203386

**Published:** 2018-09-07

**Authors:** Masahiro Nakatochi, Yingsong Lin, Hidemi Ito, Kazuo Hara, Fumie Kinoshita, Yumiko Kobayashi, Hiroshi Ishii, Masato Ozaka, Takashi Sasaki, Naoki Sasahira, Manabu Morimoto, Satoshi Kobayashi, Makoto Ueno, Shinichi Ohkawa, Naoto Egawa, Sawako Kuruma, Mitsuru Mori, Haruhisa Nakao, Chaochen Wang, Takeshi Nishiyama, Takahisa Kawaguchi, Meiko Takahashi, Fumihiko Matsuda, Shogo Kikuchi, Keitaro Matsuo

**Affiliations:** 1 Division of Data Science, Data Coordinating Center, Department of Advanced Medicine, Nagoya University Hospital, Nagoya, Japan; 2 Department of Public Health, Aichi Medical University School of Medicine, Nagakute, Japan; 3 Division of Cancer Information and Control, Aichi Cancer Center Research Institute, Nagoya, Japan; 4 Department of Gastroenterology, Aichi Cancer Center Hospital, Nagoya, Japan; 5 Clinical Research Center, National Hospital Organization Shikoku Cancer Center, Matsuyama, Japan; 6 Department of Hepato-biliary-pancreatic Medicine, The Cancer Institute Hospital of Japanese Foundation for Cancer Research, Tokyo, Japan; 7 Hepatobiliary and Pancreatic Medical Oncology Division, Kanagawa Cancer Center Hospital, Kanagawa, Japan; 8 Tokyo Metropolitan Hiroo Hospital, Tokyo, Japan; 9 Department of Internal Medicine, Tokyo Metropolitan Komagome Hospital, Tokyo, Japan; 10 Hokkaido Chitose College of Rehabilitation, Hokkaido, Japan; 11 Division of Hepatology and Pancreatology, Aichi Medical University School of Medicine, Nagakute, Japan; 12 Department of Public Health, Nagoya City University Graduate School of Medicine, Nagoya, Japan; 13 Center for Genomic Medicine, Graduate School of Medicine, Kyoto University, Kyoto, Japan; 14 Division of Cancer Epidemiology and Prevention, Aichi Cancer Center Research Institute, Nagoya, Japan; Ohio State University Wexner Medical Center, UNITED STATES

## Abstract

Genome-wide association studies (GWASs) have identified many single nucleotide polymorphisms (SNPs) that are significantly associated with pancreatic cancer susceptibility. We sought to replicate the associations of 61 GWAS-identified SNPs at 42 loci with pancreatic cancer in Japanese and to develop a risk model for the identification of individuals at high risk for pancreatic cancer development in the general Japanese population. The model was based on data including directly determined or imputed SNP genotypes for 664 pancreatic cancer case and 664 age- and sex-matched control subjects. Stepwise logistic regression uncovered five GWAS-identified SNPs at five loci that also showed significant associations in our case-control cohort. These five SNPs were included in the risk model and also applied to calculation of the polygenic risk score (PRS). The area under the curve determined with the leave-one-out cross-validation method was 0.63 (95% confidence interval, 0.60–0.66) or 0.61 (0.58–0.64) for versions of the model that did or did not include cigarette smoking and family history of pancreatic cancer in addition to the five SNPs, respectively. Individuals in the lowest and highest quintiles for the PRS had odds ratios of 0.62 (0.42–0.91) and 1.98 (1.42–2.76), respectively, for pancreatic cancer development compared with those in the middle quintile. We have thus developed a risk model for pancreatic cancer that showed moderately good discriminatory ability with regard to differentiation of pancreatic cancer patients from control individuals. Our findings suggest the potential utility of a risk model that incorporates replicated GWAS-identified SNPs and established demographic or environmental factors for the identification of individuals at increased risk for pancreatic cancer development.

## Introduction

Pancreatic cancer is a malignancy characterized by an elusive etiology, poor prognosis, and a lack of effective early detection tools. In Japan, pancreatic cancer represents the fourth leading cause of cancer deaths, with age-adjusted incidence and mortality rates having increased continuously over the past several decades[1]. The reason for this increasing pancreatic cancer burden is unclear, but it does not appear to be explained by risk factors, such as age and cigarette smoking, that have been established on the basis of epidemiologic studies[2].

The heritability of pancreatic cancer in Scandinavia has been estimated to be 36% by twin studies[3], suggestive of an important contribution of genetic variation to pancreatic cancer susceptibility. Focusing on common genetic variation represented by single nucleotide polymorphisms (SNPs), the National Cancer Institute Cohort Consortia (PanSCan) have conducted four genome-wide association studies (GWASs) with populations of European ancestry and identified 18 risk loci that were robustly associated (*P* < 5 × 10^−8^) with pancreatic cancer risk[4–9]. GWASs in two populations (Japanese and Chinese) of East Asian ancestry identified additional risk loci that subsequently failed replication in other independent cohorts[10–12]. Whereas GWASs have improved our understanding of the role of common SNPs in pancreatic cancer, the identified SNPs confer relatively small increments in risk (1.1- to 1.5-fold) with regard to the development of this disease. Furthermore, it remains a challenge to identify low-frequency or rare variants and to further clarify their contribution to pancreatic cancer pathophysiology.

Detection of pancreatic cancer at an early stage in the general population is difficult. The relatively low prevalence of the condition, coupled with the lack of validated biomarkers or imaging modalities, has limited the feasibility of a population-based screening program. Such challenges can, in part, be addressed by the development of a risk prediction model that aims to identify a small subset of high-risk individuals by incorporating genetic variants as well as demographic and lifestyle risk factors[13]. As with risk prediction models for other cancer types, such as lung, breast, and colorectal cancer[14–18], the clinical utility of such risk models for pancreatic cancer is currently limited.

As far as we are aware, no risk model has been developed for the prediction of pancreatic cancer risk in the general population in Japan. As a first step toward the development of such a model, we examined SNPs identified by published GWASs for their associations with pancreatic cancer risk in an age- and sex-matched case-control data set of Japanese individuals. We then developed the model further by incorporating the GWAS-identified SNPs that showed significant associations in our case-control cohort as well as demographic and established risk factors. A validated risk model may help to identify individuals at increased risk for the development of pancreatic cancer, thereby raising awareness that can result in the adoption of risk-minimizing behavior or in early detection by follow-up examinations.

## Materials and methods

### Study subjects

To develop the risk model, we performed a genetic association study with 664 cases and 664 age- and sex-matched controls selected from two case-control data sets that included a total of 945 pancreatic cancer patients and 2109 control subjects. The first data set included 622 patients who were recruited for a multi-institutional case-control study of pancreatic cancer, the details of which have been described previously[19]. In this previous study, clinically or histologically (or both) diagnosed pancreatic cancer patients were recruited from January 2010 to July 2014 at five participating hospitals in central Japan, Kanto, and Hokkaido regions. Most of the patients were recruited by gastroenterologists and had tumors at stage 3 or stage 4 at the time of diagnosis. Approximately 33% of the patients underwent surgical resection. Questionnaire data on demographic and lifestyle factors and 7-ml blood samples were collected from the study participants. The second data set included 323 newly diagnosed pancreatic cancer patients as well as 2109 control subjects who were recruited to an epidemiologic research program at Aichi Cancer Center (HERPACC). All new outpatients at Aichi Cancer Center on their first visit were invited to participate in HERPACC. Those who agreed to participate filled out a self-administered questionnaire and provided a 7-ml blood sample. The data collected were entered into the HERPACC database and linked to the hospital cancer registry system periodically to confirm cancer diagnoses. The feasibility of using first-visit outpatients as control subjects within the framework of HERPACC has been addressed previously by comparing their epidemiologic features with those of randomly selected individuals from the general population[20]. In these two case-control data sets, the vast majority of pancreatic cancer cases had a histology of ductal adenocarcinoma, with a small proportion of endocrine tumors (1.7%) also being included. None of the control subjects had a diagnosis of cancer at the time of recruitment. For all case and control subjects in the present study, data on demographic and lifestyle factors, such as cigarette smoking and family history of pancreatic cancer in first-degree relatives, were extracted from questionnaire answers. Written informed consent was obtained from all study participants, and the study protocol was approved by the ethical board of Aichi Medical University, the institutional ethics committee of Aichi Cancer Center, the Human Genome and Gene Analysis Research Ethics Committee of Nagoya University, and the ethics committees of all participating hospitals.

For the present case-control genetic association study, cases diagnosed with endocrine tumors were excluded. Case and control subjects were matched according to sex and age (categorized in 5-year intervals). During the matching process, only individuals with available data for all variables, including age, sex, cigarette smoking status, and family history of pancreatic cancer, were selected. Totals of 664 cases and 664 control subjects were eligible for statistical analysis.

### Genotyping and quality control

A total of 945 pancreatic cancer case subjects and 2109 control subjects were genotyped at the Center for Genomic Medicine, Kyoto University, with the use of a HumanCoreExome-12 v1.1 BeadChip array (Illumina, San Diego, CA, USA). Five samples with a genotype call rate of <0.98 were excluded. No samples showed a discrepancy between genetic and reported sex. The identity-by-descent method implemented in PLINK 1.9 software[21] detected 17 duplicate or closely related pairs of samples (pi-hat > 0.1875), with one sample of each pair being excluded. Principal component analysis (PCA)[22] with the 1000 Genomes Project reference panel (phase 3)[23] detected seven subjects with estimated ancestries outside of the Japanese population. These seven samples were also excluded. Furthermore, PCA based on only our samples was performed to identify population outliers. On the basis of the first 10 principal components, nine population outliers were identified and were excluded from further analysis. Among the 542,585 SNPs that were genotyped with the array, we excluded nonautosomal SNPs as well as SNPs with a genotype call rate of <0.98 or a Hardy-Weinberg equilibrium exact test *P* value of <1 × 10^−6^ in the control subjects, a minor allele frequency of <0.01, or a departure from the allele frequency computed from the 1000 Genomes Project phase 3 EAS samples. Such quality control filtering resulted in the selection of 942 case subjects and 2074 control subjects as well as 248,185 SNPs.

### Genotype imputation and postimputation processing

Genotype imputation was performed with SHAPEIT2[24] and Minimac3[25] software based on the 1000 Genomes Project cosmopolitan reference panel (phase 3)[23]. We searched GWAS Catalog[26] for published GWASs of pancreatic cancer and selected 77 candidate SNPs at 54 loci that had been characterized and found to be associated with pancreatic cancer (S1 Table). Of these 77 SNPs, 16 polymorphisms at 14 loci with an imputation quality score (*r*^2^) of <0.8 (rs1747924, rs351365, rs4927850, rs35226131, rs6879627, rs73328514, rs6971499, rs10094872, rs1886449, rs7190458, rs7200646, rs4795218, rs77038344, rs11655237, rs7214041, rs6073450) were excluded. After imputation, we were finally left with 664 case subjects and 664 age- and sex-matched control subjects as well as 61 SNPs at 42 loci for statistical analysis.

### Statistical analysis

To build a high-precision risk model, we applied a three-step approach. We initially performed a screening analysis with the 61 SNPs at 42 loci in which the cutoff *P* value was defined as <0.05 for logistic regression analysis. After this screening analysis and exclusion of SNPs in strong linkage disequilibrium with other polymorphisms, eight SNPs at seven loci that were significantly associated with pancreatic cancer remained for identification of SNPs that independently influence pancreatic cancer by logistic regression analysis with a stepwise forward selection procedure. Finally, we constructed two versions of a prediction model for pancreatic cancer: Model A included established risk factors and the five SNPs at five loci identified in the stepwise selection, and model B included only the SNPs.

Simple comparison of demographic and lifestyle risk factors between case and control groups was carried out with Fisher’s exact test and Student’s *t* test. In the screening step, the association between each SNP and pancreatic cancer was assessed with the use of logistic regression analysis. We used imputed genotype, which is the expected number of risk alleles for pancreatic cancer and is a continuous variable ranging from 0 to 2. We applied two types of analysis condition to assess the association of each SNP with pancreatic cancer: condition 1, in which no covariates were included, and condition 2, in which covariates comprised smoking status (nonsmoker = 0, ever-smoker = 1) and family history of pancreatic cancer (no = 0, yes = 1). In the subsequent step, multiple logistic regression analysis with a stepwise forward selection procedure was performed to identify SNPs that independently contribute to pancreatic cancer; the dependent variable was pancreatic cancer status (control = 0, case = 1) and independent variables included the imputed genotypes of each SNP. The significance level for inclusion in and exclusion from the model construction was *P* < 0.05. A version of the model including classical risk factors and SNPs identified by the stepwise selection was designated model A, whereas a version including only the five identified SNPs was designated model B. Receiver operating characteristic (ROC) analysis with the leave-one-out cross-validation (LOOCV) method was applied to evaluate model performance with the use of pROC of the R package[27]. Confidence intervals for area under the curve (AUC) values were assessed by 10,000-times bootstrap resampling.

We defined the polygenic risk score (PRS) for pancreatic cancer as the summation of the number of risk alleles multiplied by the corresponding natural logarithm of the odds ratio, ln(OR), in model B as follows:

$$PRS=\sum_{i}^{m}ln({OR}_{i})\times x_{i}$$

where *m* is the number of SNPs (*m* = 5 in this study), OR_*i*_ is the odds ratio for SNP *i* in model B, and *x*_*i*_ is the genotype coded as the number of risk alleles for SNP *i*. We calculated the PRS for each subject using this equation and then divided the study subjects into quintile groups (Q1 to Q5) with equal numbers of control subjects on the basis of the PRS. We compared the middle quintile group (Q3) with other groups (Q1, Q2, Q4, Q5) with the use of logistic regression analysis with adjustment for cigarette smoking and family history of pancreatic cancer.

Heritability analysis was performed with the use of GCTA software[28]. The analysis estimates the percentage of phenotypic variance explained by common SNPs. We assumed a prevalence of 0.000095 for pancreatic cancer in the Japanese population on the basis of data in the GLOBOCAN 2012 database[29]. To estimate the heritability, we used the data set comprising the 664 cases and 664 controls adopted for the association analysis as well as the 248,185 directly genotyped SNPs used for imputation.

A *P* value of <0.05 was considered statistically significant. All statistical analysis was performed with SAS software version 9.4 (SAS Institute, Cary, NC, USA) and the R project version 3.3 (www.r-project.org).

## Results

We performed genotyping for 945 pancreatic cancer case subjects and 2109 control subjects with the use of a HumanCoreExome-12 v1.1 BeadChip array and imputed genotypes based on the 1000 Genomes Project cosmopolitan reference panel (phase 3). We picked up 77 candidate SNPs at 54 loci that had been characterized and found to be associated with pancreatic cancer in previous GWASs (S1 Table). Of these 77 SNPs, 16 SNPs at 14 loci were excluded from further analysis because of poor imputation quality. After postimputation processing, 664 case subjects and 664 age- and sex-matched control subjects as well as 61 SNPs at 42 loci remained for the subsequent analysis.

The demographic and lifestyle risk factors for the case and control data set selected for development of the risk model are shown in Table 1. The mean age was 60.8 years for the case subjects and 60.5 years for the controls (*P* = 0.453). Case subjects had a higher proportion of individuals with a family history of pancreatic cancer (6.0% versus 2.3%, *P* < 0.001) and ever-smokers (65.7% versus 53.0%, *P* < 0.001) compared with controls.

**Table 1:** Characteristics of case and control subjects.

Characteristic	Controls (*n* = 664)	Cases (*n* = 664)	*P* value
Age (years)	60.5 ± 8.3	60.8 ± 8.3	0.453
Male	440 (66.3%)	440 (66.3%)	1.000
Height (cm)	162.2 ± 8.4	163.5 ± 8.2	0.003
Weight (kg)	60.9 ± 10.0	61.2 ± 11.4	0.683
Body mass index (kg/m^2^)	23.1 ± 2.8	22.8 ± 3.3	0.076
Ever-smoker	352 (53.0%)	436 (65.7%)	<0.001
Family history of pancreatic cancer	15 (2.3%)	40 (6.0%)	<0.001

Continuous data are means ± s.d. Differences in continuous or noncontinuous variables between case and control groups were evaluated with Student’s *t* test or Fisher’s exact test, respectively.

**Fig 1:**
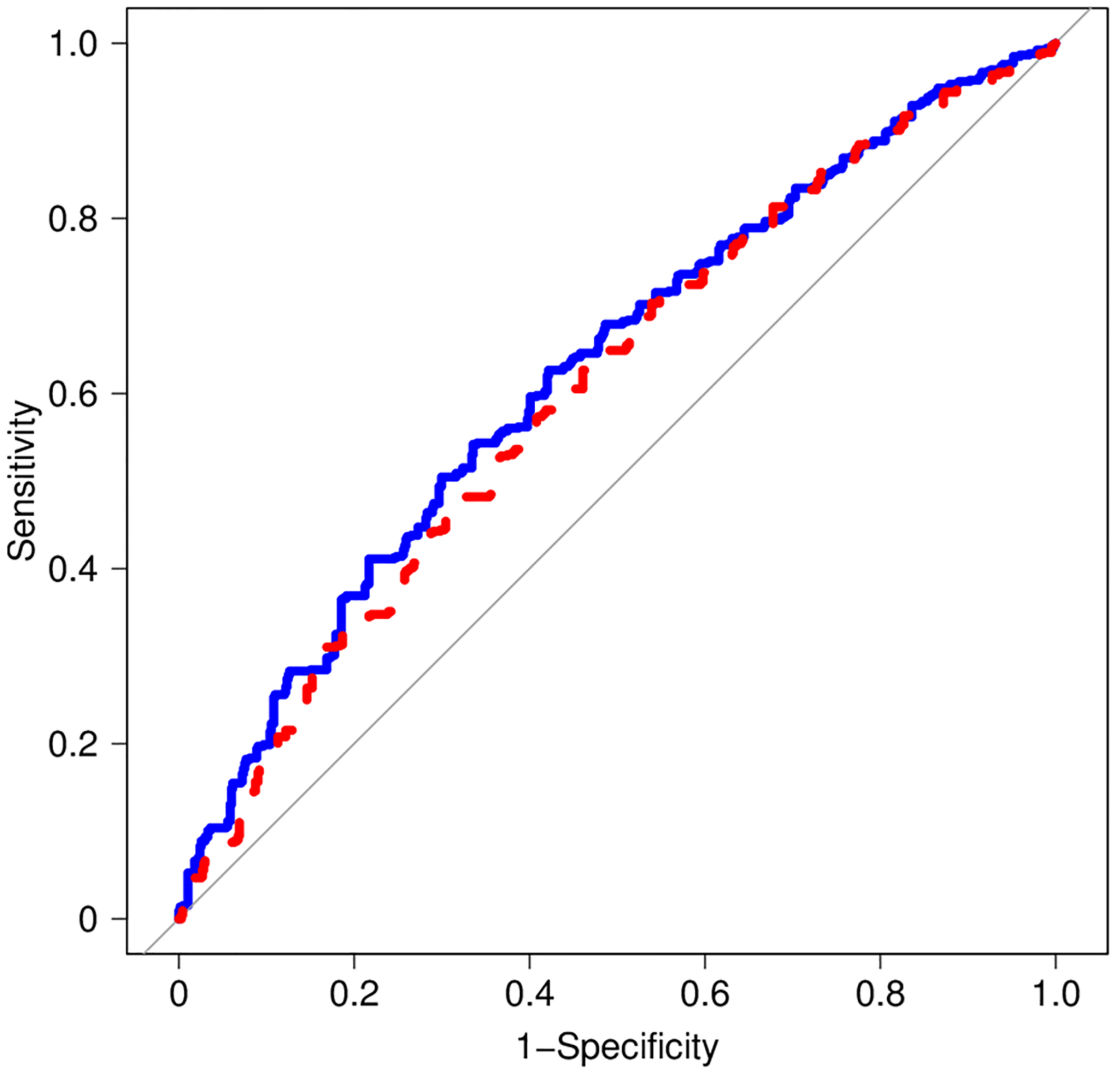
ROC curves for models A and B incorporating different variables according to the LOOCV method. Model A (blue line) incorporates classical risk factors and five GWAS-identified SNPs, whereas model B (red dashed line) includes only the five GWAS-identified SNPs. AUC values (95% CI) for models A and B are 0.63 (0.60–0.66) and 0.61 (0.58–0.64), respectively. The gray diagonal line corresponds to an AUC of 0.5 and no discrimination.

Thirteen SNPs at seven loci of the remaining 61 SNPs showed a significant association with pancreatic cancer in our case-control data set with a *P* value of <0.05 in condition 1 with no covariate or in condition 2 with the covariates of smoking status and family history of pancreatic cancer (Table 2, S2 Table). The OR for these 13 SNPs ranged from 1.19 to 1.43 for individuals with risk variants. One polymorphism of each SNP pair that exhibited strong linkage disequilibrium (*r*^2^ > 0.8) was excluded, leaving eight SNPs at seven loci for stepwise logistic regression analysis. Five SNPs, cigarette smoking, and family history of pancreatic cancer remained significantly associated with pancreatic cancer risk in the stepwise logistic regression analysis and were therefore included in the development of two versions of the risk prediction model: Model A included cigarette smoking, family history of pancreatic cancer, and the five SNPs at five loci (Table 3), whereas model B included only the five SNPs (S3 Table). Akaike information criterion values for models A and B were 1764 and 1796, respectively. In model A, ever-smokers had a 1.5-fold increased risk compared with nonsmokers (OR = 1.58, with a 95% confidence interval [CI] of 1.26–1.98). The OR for individuals with the effect alleles ranged from 1.20 to 1.43, after adjustment for cigarette smoking and family history of pancreatic cancer. The AUC values for the ROC curves derived from models A and B with the use of the LOOCV method were 0.63 (95% CI, 0.60–0.66) and 0.61 (0.58–0.64), respectively (Fig 1).

**Table 2:** Association of pancreatic cancer with 13 SNPs at seven loci in the case-control data set with a *P* value of <0.05.

SNP	Locus	Position	Nearby genes	Alleles	Risk allele	RAF		Condition 1		Condition 2	
						Case	Control	OR (95% CI)	*P* value	OR (95% CI)	*P* value
rs13303010	1p36.33	894573	*NOC2L*	G/A	G	0.311	0.274	1.19 (1.01–1.41)	0.039	1.20 (1.01–1.42)	0.034
rs12615966	2q12.1	105378957	*LINC01114*, *LINC01158*	C/T	T	0.113	0.089	1.34 (1.03–1.74)	0.032	1.30 (1.00–1.70)	0.053
rs12478462	2q23.3	153654720	*ARL6IP6*, *RPRM*	T/G	G	0.687	0.648	1.19 (1.01–1.40)	0.037	1.19 (1.01–1.40)	0.039
rs9854771	3q28	189508471	*TP63*	G/A	G	0.913	0.889	1.30 (1.00–1.68)	0.046	1.27 (0.98–1.64)	0.072
rs687289	9q34.2	136137106	*ABO*	G/A	A	0.520	0.437	1.43 (1.22–1.67)	<0.001	1.42 (1.21–1.66)	<0.001
rs657152	9q34.2	136139265	*ABO*	C/A	A	0.508	0.426	1.42 (1.21–1.66)	<0.001	1.41 (1.20–1.65)	<0.001
rs505922	9q34.2	136149229	*ABO*	T/C	C	0.511	0.438	1.36 (1.16–1.60)	<0.001	1.36 (1.16–1.60)	<0.001
rs630014	9q34.2	136149722	*ABO*	A/G	G	0.669	0.627	1.21 (1.03–1.43)	0.019	1.21 (1.03–1.43)	0.022
rs9564966	13q22.1	73896221	*KLF5*, *LINC00392*	A/G	A	0.558	0.469	1.42 (1.22–1.65)	<0.001	1.40 (1.20–1.63)	<0.001
rs9573163	13q22.1	73908846	*KLF5*, *LINC00392*	C/G	C	0.558	0.470	1.42 (1.22–1.65)	<0.001	1.40 (1.20–1.64)	<0.001
rs4885093	13q22.1	73910026	*KLF5*, *LINC00392*	G/A	G	0.565	0.483	1.39 (1.19–1.62)	<0.001	1.37 (1.18–1.60)	<0.001
rs9543325	13q22.1	73916628	*KLF5*, *LINC00392*	C/T	C	0.562	0.475	1.40 (1.21–1.63)	<0.001	1.39 (1.19–1.62)	<0.001
rs16986825	22q12.1	29300306	*ZNRF3*	C/T	T	0.521	0.467	1.24 (1.07–1.44)	0.005	1.22 (1.05–1.42)	0.011

Risk alleles are defined in S1 Table with the exception of rs687289, the risk alleles for which are based on our data set. OR and *P* values in condition 2 were adjusted for cigarette smoking and family history of pancreatic cancer. OR values represent increased risk of pancreatic cancer per risk allele copy for each SNP. RAF, risk allele frequency.

**Table 3:** Demographic and lifestyle risk factors as well as the five SNPs included in risk model A.

Factor or SNP	Locus	Position	Alleles	Risk allele	OR (95% CI)	*P* value
Ever-smoker					1.58 (1.26–1.98)	<0.001
Family history of pancreatic cancer					2.61 (1.41–4.86)	0.002
rs13303010	1p36.33	894573	G/A	G	1.20 (1.01–1.42)	0.039
rs12615966	2q12.1	105378957	C/T	T	1.32 (1.01–1.74)	0.045
rs657152	9q34.2	136139265	C/A	A	1.43 (1.22–1.69)	<0.001
rs9564966	13q22.1	73896221	A/G	A	1.42 (1.21–1.66)	<0.001
rs16986825	22q12.1	29300306	C/T	T	1.22 (1.04–1.42)	0.014

Risk alleles are defined in S1 Table. OR represents increased risk of pancreatic cancer per risk allele copy for each SNP.

**Fig 2:**
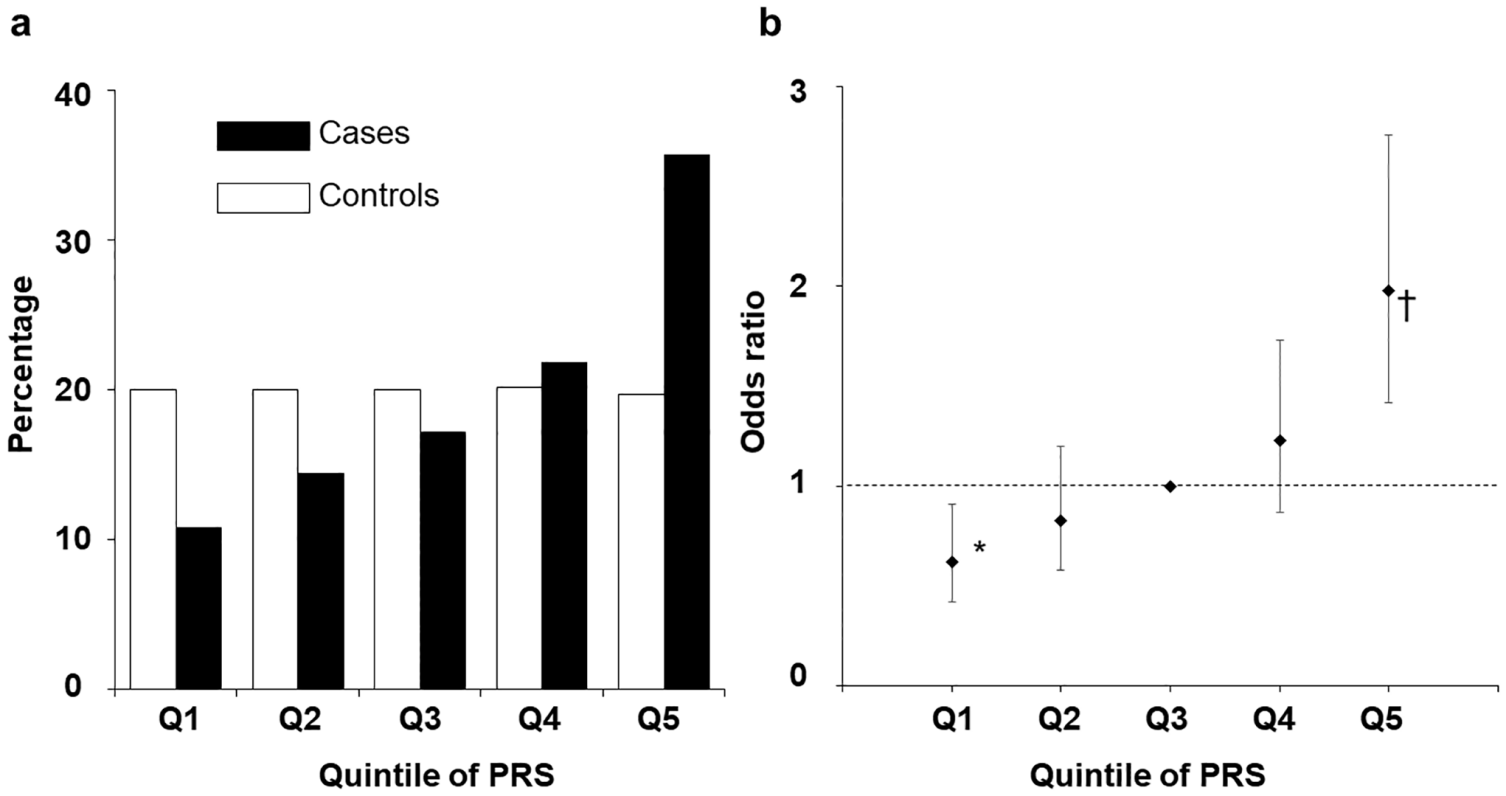
Percentage of subjects as well as the OR for pancreatic cancer according to PRS. (**a**) Distribution of the PRS in pancreatic cancer case and control subjects. (**b**) The OR for pancreatic cancer according to the quintiles of PRS. Vertical bars represent 95% CIs. The horizontal dashed line indicates the null value (OR = 1.0). *P < 0.05, †*P* < 0.01 versus Q3. The cutoffs for the quintiles of PRS in the control subjects were Q1 ≤ 0.58, 0.58 < Q2 ≤ 0.91, 0.91 < Q3 ≤ 1.12, 1.12 < Q4 ≤ 1.31, and Q5 > 1.31. OR values were calculated by logistic regression analysis with adjustment for cigarette smoking and family history of pancreatic cancer.

We calculated the polygenic risk score (PRS) for each study subject using model B and then divided the subjects into quintile groups (Q1 to Q5) with equal numbers of control individuals on the basis of the PRS (Fig 2a). The mean ± s.d. values of the PRS for case and control subjects were 1.17 ± 0.42 and 1.01 ± 0.42, respectively. The PRS was significantly associated with risk of pancreatic cancer (Fig 2b). Compared with subjects in the middle quintile of PRS values (Q3), the OR values were 0.62 (95% CI, 0.42–0.91), 0.83 (0.58–1.20), 1.23 (0.87–1.73), and 1.98 (1.42–2.76) for subjects in Q1, Q2, Q4, and Q5, respectively, after adjustment for cigarette smoking and family history of pancreatic cancer.

Finally, we estimated the heritability of pancreatic cancer due to common GWAS SNPs using only data for directly genotyped SNPs (248,185 SNPs for 664 cases and 664 age- and sex-matched controls). For a disease prevalence of 0.000095, we estimated that 16.1% (95% CI, 7.8–24.3%) of the total phenotypic variation in our data set was explained by common SNPs across the genome.

## Discussion

Risk prediction models incorporating SNPs and environmental risk factors offer a means to identify a subset of individuals with increased cancer risk in the general population[30]. With the use of a case-control data set based on the Japanese population, we have now developed a risk model for pancreatic cancer and showed that it performed moderately well for discrimination of pancreatic cancer patients from individuals without the disease. Our findings suggest that a risk model that incorporates replicated GWAS-identified SNPs and established environmental factors is potentially useful for the identification of a subset of Japanese individuals with an increased risk for the development of pancreatic cancer.

Three risk models have been developed to date for the prediction of pancreatic cancer risk in general populations of different ethnicities[13,31,32]. To identify individuals at elevated risk for pancreatic cancer in a population of European ancestry, Klein et al. estimated the absolute risk of pancreatic cancer development with a risk model (based on three GWAS-identified SNPs, sex, age, ABO genotype, family history of pancreatic cancer, body mass index, cigarette smoking, and heavy alcohol intake) and incidence data from the SEER registries[13]. The AUC for the model was 0.61 (95% CI, 0.58–0.63), which demonstrated its superiority over a model that included only genetic or only nongenetic factors. However, only a few individuals were estimated to have a 10-year absolute risk of >2% even if all genetic and nongenetic factors were present, indicating that the clinical utility of the model is low. By combining SEER data with a logistic model including risk factors (cigarette smoking, current use of proton pump inhibitors, recent diagnosis of diabetes mellitus and pancreatitis, Jewish ancestry, and ABO blood group other than O) that were identified from a population-based case-control study, Risch et al.[31] showed that 0.87% of controls with a combination of risk factors had an estimated 5-year absolute risk of >5%. It should be noted that these two models were developed for populations of European ancestry and that their performance in populations of Asian ancestry, including Japanese, awaits validation. With regard to East Asian populations, Yu et al. developed a risk prediction model to estimate individual risk of pancreatic cancer in the Korean population[32]. The model included biomarkers such as fasting blood glucose and urinary glucose levels as well as demographic and lifestyle risk factors, and it showed good discrimination ability with a validation set, with C-statistics of 0.81 (95% CI, 0.80–0.83) for men and 0.80 (0.79–0.82) for women. No genetic factors were included in this prediction model, however. The discrimination ability of our risk model is similar to that of the model of Klein et al.[13], and it would be similar to that of the model of Yu et al.[32] for the Korean population if we included matching factors such as age and sex. However, one key issue with all these risk models is the difficulty of their translation to clinical or public health practice. Further studies are thus needed to clarify the application of risk prediction models in different contexts, such as for population-wide use as a risk assessment tool or for screening for individuals with a high absolute risk of pancreatic cancer.

The PRS is independent of established risk factors and can provide risk stratification beyond family history[33]. Given that a polygenic component to pancreatic cancer risk was suggested by previous studies[34], we calculated the PRS using the five replicated GWAS-identified SNPs. Our results showed that this approach provided a good stratification of pancreatic cancer risk. Exploration of the polygenic contribution to pancreatic cancer risk beyond the known risk variants, however, will require studies with larger sample sizes and more sophisticated analytic approaches[35]. Although our sample size limited further evaluation of the PRS, risk prediction models for pancreatic cancer that incorporate the PRS are worth pursuing, given that fewer common genetic variants have been identified for this cancer than for other cancer types such as breast and colorectal cancer.

Risk models that incorporate GWAS-identified SNPs should be interpreted in the context of heritability that can be explained by common SNPs. In the present study, we estimated that 16.1% (95% CI, 7.8–24.3%) of the total phenotypic variation in our data set was explained by common SNPs across the genome. A prediction model based on all common SNPs across the genome would thus have a performance that corresponds to the heritability. Heritability of pancreatic cancer in individuals of European descent has been estimated on the basis of common SNPs across the genome. Childs et al. estimated that 16.4% (95% CI, 10.4–22.4%) and 13.1% (95% CI, 9.9–16.3%) of the total phenotypic variation in PanC4 and in the combined data set, respectively, was explained by common SNPs across the genome[9]. Our results are thus consistent with these previous findings. The heritability of pancreatic cancer might therefore be similar in populations of Japanese or European descent.

One strength of our study is that the risk model we constructed was based on case-control data for Japanese subjects. Our risk model represents the first attempt to use existing GWAS-identified SNPs to identify a subset of the general Japanese population at increased risk of developing pancreatic cancer. We were able to replicate 13 SNPs at seven loci out of 61 GWAS-identified SNPs at 42 loci in our case-control data set. Although the effect sizes for the associations of SNPs with pancreatic cancer in our study are small, they are consistent with the results of previous GWASs for this cancer type. Furthermore, to address issues relating to overfitting, we used the LOOCV method to assess the performance of our prediction model. The AUC estimated with the LOOCV method was similar to those of previous models developed for pancreatic cancer, supporting the validity of our risk model based on the selected SNPs.

Our study also has several limitations. First, our GWAS was limited by the relatively small sample size and we did not validate our risk model in independent cohort samples. A clinically useful risk model needs to perform well with independent data sets and be generalizable to external populations. We will continue our efforts to find independent data sets with which to validate our risk model. Second, the clinical utility of our model as well as its potential contribution to a reduction in pancreatic cancer mortality in the general population are limited, although the discriminatory ability of the version of the model that included both demographic and lifestyle factors and replicated GWAS-identified SNPs was better than that of the version based only on SNPs. As shown previously[13], risk models that focus on rare cancer types such as pancreatic cancer may not offer clinically meaningful risk stratification because the percentage of individuals with a high absolute risk who warrant follow-up examination is small. Third, although we assessed all reported genome-wide significant SNPs documented for pancreatic cancer in GWASs, it is likely that other risk variants with borderline significance were not captured. Fourth, in addition to demographic factors and family history of pancreatic cancer, our study included only cigarette smoking as the most consistent risk factor for pancreatic cancer in Japanese. Further exploration and establishment of risk factors for pancreatic cancer in Japanese subjects may contribute to refinement of risk models.

In summary, we have developed a risk model for pancreatic cancer in Japanese individuals that showed a moderately good discriminatory ability with regard to differentiation of pancreatic cancer patients from control individuals. Further research is warranted to address the clinical utility of the model or its application to population-based screening. In particular, the goal of early detection can be pursued further by incorporation of established environmental risk factors, circulating biomarkers, the PRS, as well as other “omics” data.

## Supporting information

S1 Table Information on the 77 SNPs at 54 loci extracted from published GWASs for pancreatic cancer. (DOCX)

S2 Table Association of pancreatic cancer with 61 SNPs at 42 loci extracted from published GWASs in our case-control cohort. (XLSX)

S3 Table The five SNPs at five loci included in the risk model. (XLSX)

S1 Data The data set used to construct the risk prediction model. (XLSX)
