# Supplementary material for: Prediction model for pancreatic cancer risk in the general Japanese population
Source: PLoS One. 2018 Sep 7;13(9):e0203386. doi: 10.1371/journal.pone.0203386 (PMC6128543; doi:10.1371/journal.pone.0203386)
Supplement: S1 Table — (DOCX) [file pone.0203386.s001.docx]

**S1 Table. Information on the 77 SNPs at 54 loci extracted from published GWASs for pancreatic cancer.**

| SNP | Locus | Position  (GRCh37) | Nearby genes | Alleles | Risk  allele | References | Genotyped/imputed  in our data |
| --- | --- | --- | --- | --- | --- | --- | --- |
| rs13303010 | 1p36.33 | 894573 | *NOC2L* | G/A | G | [5] | Genotyped |
| rs1747924 | 1p31.3 | 64538961 | *ROR1* | C/A | A | [9] | Imputed |
| rs351365 | 1p13.2 | 113046395 | *WNT2B* | T/C | C | [5,9] | Imputed |
| rs12029406 | 1q32.1 | 199905828 | *LINC01221, NR5A2* | C/T | C | [7] | Imputed |
| rs4465241 | 1q32.1 | 199963622 | *LINC01221, NR5A2* | T/C | T | [7] | Genotyped |
| rs10919791 | 1q32.1 | 199965168 | *LINC01221, NR5A2* | G/A | G | [7,8] | Imputed |
| rs2816938 | 1q32.1 | 199985368 | *LINC01221, NR5A2* | T/A | A | [4,5] | Imputed |
| rs3790844 | 1q32.1 | 200007432 | *NR5A2* | A/G | A | [5,7,9] | Genotyped |
| rs3790843 | 1q32.1 | 200010824 | *NR5A2* | C/T | C | [7] | Imputed |
| rs2689154 | 1q43 | 238908353 | *LINC01139, CHRM3* | C/G | G | [11] | Imputed |
| rs962856 | 2p14 | 67593803 | *LINC01828, ETAA1* | C/T | C | [9] | Genotyped |
| rs1486134 | 2p14 | 67639769 | *ETAA1, LINC01812* | G/T | G | [5,9] | Imputed |
| rs6711606 | 2q11.2 | 101922170 | *RNF149* | T/G | T | [12] | Genotyped |
| rs12615966 | 2q12.1 | 105378957 | *LINC01114, LINC01158* | C/T | T | [12] | Imputed |
| rs1427593 | 2q22.1 | 137555224 | *THSD7B* | T/C | T | [12] | Genotyped |
| rs12478462 | 2q23.3 | 153654720 | *ARL6IP6, RPRM* | T/G | G | [5] | Genotyped |
| rs6736997 | 2q37.2 | 235615197 | *LINC01173* | A/C | A | [12] | Genotyped |
| rs9854771 | 3q28 | 189508471 | *TP63* | G/A | G | [5,9] | Genotyped |
| rs4927850 | 3q29 | 195751630 | *SDHAP1, TFRC* | T/C | T | [11] | Imputed |
| rs6537481 | 4q31.22 | 148396094 | *TTC29, EDNRA* | A/G | A | [5] | Imputed |
| rs2736098 | 5p15.33 | 1294086 | *TERT* | C/T | C | [5,8] | Imputed |
| rs35226131 | 5p15.33 | 1295373 | *TERT* | C/T | C | [4,5] | Imputed |
| rs401681 | 5p15.33 | 1322087 | *CLPTM1L* | C/T | T | [5,7,9] | Genotyped |
| rs31490 | 5p15.33 | 1344458 | *CLPTM1L* | G/A | ? ^a^ | [8] | Imputed |
| rs6879627 | 5p15.33 | 2109901 | *CTD-2194D22.4,*  *LOC100506858* | T/C | C | [12] | Imputed |
| rs2255280 | 5p13.1 | 39394989 | *DAB2* | C/A | A | [11] | Genotyped |
| rs9502893 | 6p25.3 | 1340189 | *FOXQ1, FOXF2* | C/T | C | [12] | Genotyped |
| rs9363918 | 6q12 | 69142008 | *LOC101928280,*  *LOC101928307* | T/G | T | [11] | Genotyped |
| rs4269383 | 6q25.3 | 156197502 | *LOC105378068,MIR1202* | A/G | G | [11] | Genotyped |
| rs3016539 | 6q26 | 162236075 | *PRKN* | C/T | T | [12] | Genotyped |
| rs17688601 | 7p14.1 | 40866663 | *SUGCT* | C/A | C | [5,9] | Genotyped |
| rs73328514 | 7p12.3 | 47488569 | *TNS3* | A/T | A | [5] | Imputed |
| rs6971499 | 7q32.3 | 130680521 | *LINC-PINT* | T/C | T | [5,8,9] | Imputed |
| rs7779540 | 7q36.2 | 153622662 | *DPP6* | G/A | A | [12] | Imputed |
| rs6464375 | 7q36.2 | 153625843 | *DPP6* | C/T | T | [12] | Genotyped |
| rs6973850 | 7q36.2 | 153638248 | *DPP6* | C/T | T | [12] | Genotyped |
| rs7832232 | 8p11.22 | 38469303 | *RNF5P1, TACC1* | A/G | A | [12] | Genotyped |
| rs2941471 | 8q21.11 | 76470404 | *HNF4G* | G/A | A | [5] | Imputed |
| rs10088262 | 8q24.13 | 124765702 | *ANXA13, FAM91A1* | A/G | A | [12] | Genotyped |
| rs10094872 | 8q24.21 | 128719884 | *CASC11* | A/T | T | [4,5] | Imputed |
| rs1561927 | 8q24.21 | 129568078 | *LINC00824* | C/T | T | [5,8] | Genotyped |
| rs10974531 | 9p24.2 | 4426631 | *GLIS3, SLC1A1* | C/A | A | [11] | Genotyped |
| rs10991043 | 9q31.1 | 106797388 | *LOC101928523, SMC2-AS1* | T/C | C | [5] | Imputed |
| rs2417487 | 9q31.1 | 106887581 | *SMC2* | A/G | A | [5] | Imputed |
| rs687289 | 9q34.2 | 136137106 | *ABO* | G/A | ? ^a^ | [8] | Genotyped |
| rs657152 | 9q34.2 | 136139265 | *ABO* | C/A | A | [6] | Genotyped |
| rs505922 | 9q34.2 | 136149229 | *ABO* | T/C | C | [5,6,9] | Genotyped |
| rs630014 | 9q34.2 | 136149722 | *ABO* | A/G | G | [6] | Genotyped |
| rs12413624 | 10q26.11 | 120278944 | *LINC00867, PRLHR* | T/A | T | [11] | Genotyped |
| rs708224 | 12p11.21 | 32436409 | *BICD1* | A/G | A | [12] | Genotyped |
| rs7310409 | 12q24.31 | 121424861 | *HNF1A* | A/G | A | [9] | Genotyped |
| rs1182933 | 12q24.31 | 121454622 | *C12orf43* | C/T | T | [5] | Imputed |
| rs9554197 | 13q12.2 | 28476978 | *PLUT* | C/T | T | [9] | Genotyped |
| rs9581943 | 13q12.2 | 28493997 | *PDX1* | G/A | A | [5,8,9] | Imputed |
| rs1585440 | 13q21.32 | 66481815 | *LINC01052, MIR548X2* | G/T | G | [12] | Genotyped |
| rs9564966 | 13q22.1 | 73896221 | *KLF5, LINC00392* | A/G | A | [7] | Genotyped |
| rs9573163 | 13q22.1 | 73908846 | *KLF5, LINC00392* | C/G | C^b^ | [11] | Imputed |
| rs4885093 | 13q22.1 | 73910026 | *KLF5, LINC00392* | G/A | G | [11] | Imputed |
| rs9543325 | 13q22.1 | 73916628 | *KLF5, LINC00392* | C/T | C | [5,7,8,9] | Genotyped |
| rs1886449 | 13q22.1 | 73932114 | *KLF5, LINC00392* | C/T | T | [12] | Imputed |
| rs2039553 | 13q31.1 | 80299722 | *LINC01068, LINC01038* | A/G | A | [12] | Genotyped |
| rs7190458 | 16q23.1 | 75263661 | *BCAR1* | G/A | A | [5,8] | Imputed |
| rs7200646 | 16q24.1 | 86335351 | *LINC02135, LINC00917* | C/T | C | [5] | Imputed |
| rs4924935 | 17p11.2 | 18753870 | *TVP23B, PRPSAP2* | C/T | C | [12] | Genotyped |
| rs225190 | 17q11.2 | 30877658 | *MYO1D* | C/T | C | [12] | Genotyped |
| rs4795218 | 17q12 | 36078510 | *HNF1B* | G/A | G | [5] | Imputed |
| rs77038344 | 17q21.2 | 38644214 | *TNS4* | C/T | T | [5] | Imputed |
| rs2257205 | 17q22 | 56448297 | *RNF43* | C/T | T | [12] | Genotyped |
| rs11655237 | 17q24.3 | 70400166 | *LINC00673* | C/T | T | [9] | Imputed |
| rs7214041 | 17q24.3 | 70401476 | *LINC00673* | C/T | T | [5,9] | Imputed |
| rs1517037 | 18q21.32 | 56878274 | *SEC11C, GRP* | C/T | C | [5,9] | Genotyped |
| rs6073450 | 20q13.12 | 43086648 | *LINC01620* | G/A | A | [5,9] | Imputed |
| rs372883 | 21q21.3 | 30717737 | *BACH1* | T/C | T | [11] | Genotyped |
| rs1547374 | 21q22.3 | 43778895 | *TFF2, TFF1* | A/G | A | [11] | Genotyped |
| rs450960 | 22q11.21 | 18316304 | *MICAL3* | C/T | T | [5] | Imputed |
| rs16986825 | 22q12.1 | 29300306 | *ZNRF3* | C/T | T | [5,8] | Genotyped |
| rs5768709 | 22q13.32 | 48929569 | *FAM19A5* | A/G | G | [11] | Imputed |

Risk allele indicates the allele reported to be associated with increased risk for pancreatic cancer in the published study. ^a^Risk allele not reported in the reference. ^b^Risk allele for rs9573163 is reported to be the G allele in the reference; however, the only strand containing the SNP is reversed according to nearby SNPs such as rs9564966, rs4885093, and rs9543325, and we therefore set the C allele as the risk allele.
